# Head and Spinal Injuries in Pediatrics: Descriptive Study over 10 Years in a Tertiary Hospital

**DOI:** 10.7759/cureus.74832

**Published:** 2024-11-30

**Authors:** Liqaa Raffee, Dania Al Miqdad, Khaled Alawneh, Nour Negresh, Rania Al Amaireh, Ali Al Shatnawi, Retaj Alawneh, Hasan Alawneh

**Affiliations:** 1 Department of Accident and Emergency Medicine, Jordan University of Science and Technology, Irbid, JOR; 2 Department of Accident and Emergency Medicine, King Abdullah University Hospital, Irbid, JOR; 3 Department of Diagnostic Radiology and Nuclear Medicine, Jordan University of Science and Technology, Irbid, JOR; 4 Department of Accident and Emergency Medicine, Faculty of Medicine, Al-Balqa Applied University, Al-Salt, JOR; 5 Department of Medicine, Jordan University of Science and Technology, Irbid, JOR; 6 Department of Internal Medicine, Jordanian Royal Medical Services, Amman, JOR; 7 Medical Engineering, Cardiff University School of Engineering, Wales, GBR

**Keywords:** management, morbidity, mortality, pediatric head trauma, pediatric spinal injuries

## Abstract

Objectives: Pediatric head and spinal traumas are challenging for healthcare professionals due to their potential for severe consequences. Understanding optimal management methods is crucial to prevent complications and improve outcomes. Head and spinal injuries are common in children, with falls and motor vehicle collisions as the leading causes. Common clinical features include altered mental status, vomiting, and neurological deficits. Primary injuries may involve the scalp, skull, brain, and spinal cord. Severity is classified using the Glasgow Coma Scale (GCS).

Methods: This study included pediatric patients (<18 years) presenting to the emergency department with traumatic head or spinal injuries. Data collection included patients' medical history, demographic details, trauma mechanisms, clinical presentations, treatment modalities, and laboratory findings.

Results: A total of 303 patients were analyzed, with male patients accounting for 214 (70.6%). Road traffic accidents (RTA) at 147 (48.5%) and falls at 139 (45.9%) were the most common traumas. Blunt injuries predominated, accounting for 292 cases (96.4%). The head was frequently involved 253 (83.5%). Observation was the most common treatment, used in 213 cases (70.3%), followed by intubation in 44 cases (14.5%). The mean GCS was 10.7. Most patients improved during hospitalization which stood at 272 (89.8%), with a mean length of stay of 9.02 days. Spinal trauma cases (14) showed male predominance at 12 (85.7%) and falls were the most common cause at 7 (50%). Conservative management was prevalent at 11 (78.6%), and most cases achieved survival at 13 (92.9%).

Conclusions: Prompt diagnosis and management are essential to reduce mortality and morbidity in pediatric head and spinal injuries. Accurate evaluation of injury type, location, and mechanism is crucial for effective treatment. This study highlights the importance of optimal management strategies and emphasizes the need for further research to explore factors affecting mortality and morbidity. Limitations include the small number of spinal injury cases and regional generalization.

## Introduction

Pediatric head and spinal traumas pose significant challenges to healthcare professionals due to their potential for severe and long-lasting consequences. The unique physiological and anatomical characteristics of children make them particularly vulnerable to such injuries, often resulting in significant morbidity and mortality. Thus, it is important to understand optimal management methods to prevent unnecessary immobilization and other adverse events [1].

Head and spinal injuries are common injuries among children, compromising more than half of the injuries that occur in the pediatric age group [2]. It’s most prevalent in children aged 0-4 years and adolescents aged 15-19 years. The etiology of pediatric head trauma varies according to age. Road traffic accidents (RTA) and falls account for the majority of injuries, followed by abuse and other forms of non-accidental trauma and sports-related injury [3].

Falls are the most common cause of injury and the leading cause of traumatic brain injury in children aged 0-4 years [4]. Abusive head trauma (AHT) is more common in infants under two years, with 30 out of 100,000 infants under one year hospitalized annually [5]. Male patients are more likely to receive emergency care and be hospitalized than female patients [6].

Clinical features of head trauma in pediatrics encompass a wide range of manifestations, including altered mental status, loss of consciousness, vomiting, headache, seizures, and focal neurological deficits [7].

Depending on the severity and mechanism of the trauma, patients may experience one or a combination of primary injuries including scalp injury, skull fracture, basilar skull fracture, concussion, contusion, intraventricular hemorrhage (IVH), subarachnoid hemorrhage, epidural hematoma, subdural hematoma, penetrating injuries, and diffuse axonal injury [7]. Moreover, up to 5% of patients who suffer from head injuries may also have a corresponding spinal injury, necessitating immediate medical attention. Multiple levels of the spine are involved in spinal cord injuries. The rates of incidence are cervical (55%), thoracic (15%), thoracolumbar junction (15%), and lumbosacral region (15%) [8].

Classification of the severity of head trauma is based on the GCS. A GCS of 14 to 15 indicates minor head trauma, a GCS of 9 to 13 indicates moderate head trauma, and a GCS ≤ 8 indicates severe head trauma [9].

The diagnosis and treatment options vary according to the cause and mechanism of injury. It is important first to evaluate these factors before managing the symptoms. If the patient is presented to the ER after trauma but is alert and does not show neurological defects, an X-ray is not necessary, and ruling out injuries can be done without it. However, if the patient is unconscious, suffering from pain, and/or showing some neurological defects, an X-ray is useful to diagnose the injury. In some cases, a computed tomography (CT) scan would be more appropriate, especially for ligamentous tears. Treatment options may be as simple as immobilization or as invasive as operative therapy [1,10]. The choice of treatment depends on the severity and location of the injury.

This study aims to investigate the various factors that are linked to mortality and morbidity in order to determine the most effective form of treatment, whether operative or non-operative and the best management strategy for the patients.

## Materials and methods

Study participants

We identified all pediatric patients younger than 18 years of age who presented to the emergency department (ER) in our institution from December 2011 to December 2021. All patients who presented to the ER following a traumatic injury like RTA, falling down, being hit by an object, or blast injuries were included in the study. 

Patients presenting to the ER with conditions unrelated to trauma were excluded from data collection to ensure the study focused only on traumatic injuries and their associated outcomes. Non-traumatic conditions included respiratory and cardiovascular events, neurological conditions like stroke, multiple sclerosis attacks, cerebral palsy, gynecology emergencies, and surgical non-traumatic cases such as acute appendicitis.

Data collection

Data collection for this study encompassed a comprehensive set of variables related to the patient's medical history, demographics, trauma details, and clinical presentation. The collected preoperative variables included gender, age of diagnosis, type of trauma, type of injury, trauma location, whether the injury was multiple or isolated, specific injury sites (abdomen, clavicle, chest, face, head, femur, pelvis), type of transport to the hospital, comorbidities, vital stability, presence of hemorrhage, loss of consciousness, seizure history, and the specific treatments administered. Also, the data encompassed information regarding the use of casts and chest tubes, treatment approach (conservative, intubation, Intravenous (IV) fluids, surgery, or observation), GCS score at the ER, cardiopulmonary resuscitation (CPR) performed, heart rate (HR), respiratory rate (RR), systolic and diastolic blood pressure (BP), administration of blood units within the first 24 hours, admission unit, hospital course, hospital management, length of stay in the hospital, presence of any spinal cord injuries, brain injuries, other significant injuries, overall outcome, and mental status at discharge. Furthermore, laboratory variables were collected, including international normalized ratio (INR), prothrombin time (PT), partial thromboplastin time (PTT), bicarbonate (HCO3), pH, partial pressure of carbon dioxide (pCO2), and base excess (BE). These variables provide critical information on the patient's coagulation status, acid-base balance, and respiratory function.

Ethical considerations

The study received approval from the Institutional Review Board (IRB) of King Abdullah University Hospital affiliated with Jordan University of Science and Technology. It adhered to the principles outlined in the Declaration of Helsinki, 1964. Informed consent was waived by the IRB committee due to the study's retrospective nature, as conducting the research practically necessitated the waiver. Moreover, the study posed minimal risk to patients and had no adverse impact on the rights or privacy of participants, considering the significance of the knowledge to be obtained. Patient data were anonymized, and confidentiality was upheld.

Statistical analysis

Continuous data in this study were summarized using means and standard deviations (SD), while categorical data were presented as frequencies and percentages N(%). Statistical analyses were conducted to compare continuous variables, with the student's t-test and one-way ANOVA used for normally distributed variables, and the Mann-Whitney U and one-way Kruskal-Wallis tests applied for variables that did not follow a normal distribution based on the Shapiro-Wilk test. Missing data were handled using the pairwise deletion method under the assumption of missing completely at random. Statistical significance was determined at a two-sided p-value of ≤ 0.05. The IBM SPSS Statistics for Windows, Version 26 (IBM Corp., Armonk, NY) was utilized for all data analyses.

## Results

Our analysis included 303 patients, with 214 (70.6%) being male patients and 89 (29.4%) being female patients (Table 1). The mean (SD) age of diagnosis was 6.64 (4.37), with a median of six years. The most common type of trauma was RTA at 147 (48.5%) and falling down at 139 (45.9%), while the most common type of injury was blunt at 292 (96.4%). The majority of trauma locations were on the road at 161 (53.1%), or home at 92 (30.4%). Regarding multiple or isolated injuries, 179 (58.1%) of patients had isolated injuries, while 127 (41.3%) had multiple injuries. The head was the most commonly involved site with 253 (83.5%) of patients having head injuries.

**Table 1:** Demographic and clinical characteristics of the study population. RTA: road traffic accident.

Characteristics	Overall, N=308
Gender	N (%)
-Female	89 (29.4%)
-Male	214 (70.6%)
Age of diagnosis (years)	Mean (SD): 6.64 (4.37)
Type of trauma	N (%)
-RTA	147 (48.5%)
-Falling	139 (45.9%)
-Hit by an object	10 (3.3%)
-Blast injury	4 (1.3%)
Type of injury	N (%)
-Blunt	292 (96.4%)
-Blast	4 (1.3%)
-Penetrating	3 (1.0%)
Trauma location	N (%)
-On the road	161 (53.1%)
-At home	92 (30.4%)
-Stairs	8 (2.6%)
-Victim of war	3 (1.0%)
Sites involved	N (%)
-Head	253 (83.5%)
-Abdomen	43 (14.2%)
-Face	40 (13.2%)
-Chest	40 (13.2%)
-Pelvis	15 (5.0%)
-Femur	13 (4.3%)
-Clavicle	5 (1.7%)
Type of transport	N (%)
-Ambulance	3 (1.0%)
-Private	83 (27.4%)
-Referred	147 (48.5%)
-Transferred	1 (0.3%)
Vital stability	N (%)
-Stable	254 (83.8%)
-Unstable	38 (12.5%)
Presentation	N (%)
-Hemorrhage	27 (8.9%)
-Loss of consciousness	87 (28.7%)
-Seizure	21 (6.9%)

In terms of type of transport, 147 (48.5%) of patients were referred, while 83 (27.4%) used private transport and 70 (22.8%) had an unknown transport type. Most patients, that is, 254 (83.8%) were stable upon presentation, while 38 (12.5%) were unstable. Common presentations included loss of consciousness at 87 (28.7%), hemorrhage at 27 (8.9%), and seizure at 21 (6.9%) (Table 1).

Of the 303, we evaluated 14 spinal trauma cases in this study (Table 2). Among the cases, 12 (85.7%) were male patients and two (14.3%) were female patients. The mean age of the patients was 10.98 (5.44). The most common type of trauma observed was falling down, accounting for seven (50%) cases, followed by RTA with four (28.6%) cases, and being hit by an object with three (21.4%) cases, blunt injuries were predominant in all cases. Regarding the location of the trauma, most incidents occurred at home, that is, six (42.9%), followed by streets at four (28.6%), and stairs at one (7.1%). In terms of the nature of the injury, multiple injuries were observed in 11 (78.6%) cases, while isolated injuries were present in three (21.4%) cases. Among the comorbidities, morbid obesity was noted in one (7.1%) case. Pre-hosp-vital stability was reported as stable in all cases. Hemorrhage and loss of consciousness were not commonly observed, with only a few cases showing such symptoms. Conservative management was the primary pre-hospital treatment approach for most cases 11 (78.6%). Other interventions, such as intubation, intravenous (IV) fluids, and the use of a simple face mask for O2 therapy, were also employed in some cases.

**Table 2:** Demographic and clinical characteristics of the spinal injuries patients. RTA: road traffic accident. * Data is presented as N (%) unless stated otherwise.

Characteristics	N=14^*^
Age, mean (SD)	10.98 (5.44)
Gender	N (%)
-Male	12 (85.7%)
-Female	2 (14.3%)
Type of trauma	N (%)
-Fall	7 (50%)
-RTA	4 (28.6%)
-Hit by an object	3 (21.4%)
Trauma location	N (%)
-Home	6 (42.9%)
-Street	4 (28.6%)
-Other	3 (21.4%)
-Stairs	1 (7.1%)
Nature of injury	N (%)
-Multiple injuries	11 (78.6%)
-Isolated injuries	3 (21.4%)

Upon admission to the ER, the mean GCS was 10.7 (5.77), with a median score of 14 (Table 3). Tachycardia was observed in 123 (40.6%) of patients, while tachypnea was observed in 114 (37.6%). A low diastolic BP was observed in 60 (19.8%) of patients, while a low systolic BP was observed in 49 (16.2%) of patients. The majority of patients were admitted to the neurosurgery, that is five (42.6%) patients, or the pediatric ward at two (20.8%). Most patients, that is, 272 (89.8%) showed improvement during their hospital stay, while 20 (6.6%) died and six (2.0%) deteriorated or two (0.7%) were discharged against medical advice. The mean (SD) length of stay in the hospital was 9.02 (11.3) days, with a median of six days. Most patients 213 (70.3%) received observation as their treatment. Intubation was the second most common treatment, administered to 44 (14.5%) patients. Conservative treatment was utilized in 28 (9.2%) cases, while surgery was performed on 12 (4.0%) patients. A small proportion of patients received a cast, that is, eight (2.6%), chest tube, that is, nine (3.0%), and IV fluids at three (1.0%) as part of their treatment regimen.

The patients' length of stay ranged from five to 35 days, with an average of 11.4 days (Table 3). Among the cases, spinal cord injuries were observed in five (35.7%) patients, while brain injuries were identified in four (28.6%) patients. None of the patients had any other significant injuries. The outcomes of the cases varied, with 13 (92.9%) patients achieving survival and one (7.1%) patient unfortunately succumbing to their injuries. The GCS scores upon ER admission ranged from 0 to 15, with a mean score of 11.4. Hospital management approaches differed across cases, with conservative management being employed in nine (64.3%) cases and surgery performed in five (35.7%) cases. CPR was not administered to any of the patients during their hospital stay. Blood transfusions were required for one patient (7.1%) within the first 24 hours of admission.

**Table 3:** Clinical and treatment characteristics of the study population. IV: intravenous; GCS: Glasgow Coma Scale; CPR: cardiopulmonary resuscitation; ER: emergency room; HR: heart rate; RR: respiration rate; BP: blood pressure.

Characteristics	N=303
Treatment	N (%)
-Cast	8 (2.6%)
-Chest tube	9 (3.0%)
-Conservative	28 (9.2%)
-Intubation	44 (14.5%)
-IV fluids	3 (1.0%)
-Surgery	12 (4.0%)
-Observation	213 (70.3%)
GCS in ER	Mean (SD): 10.7 (5.77)
CPR	21 (6.9%)
ER-HR	N (%)
-Bradycardia	1 (0.3%)
-Normal	179 (59.1%)
-Tachycardia	123 (40.6%)
ER-RR	N (%)
-Normal	189 (62.4%)
-Tachypnea	114 (37.6%)
ER-systolic BP	N (%)
-High	7 (2.3%)
-Low	49 (16.2%)
-Normal	247 (81.5%)
ER-diastolic BP	N (%)
-High	2 (0.7%)
-Low	60 (19.8%)
-Normal	241 (79.5%)
Any blood units given in the 24 hours	33 (10.9%)
Hospital course	N (%)
-Improved	272 (89.8%)
-Died	20 (6.6%)
-Deteriorated	6 (2.0%)
-Discharged against medical advice	2 (0.7%)
Hospital stays (days)	Mean (SD): 9.02 (11.3)
Missing	6 (2.0%)

Table 4 summarizes the INR, PT, PTT, HCO3, pH, pCO2, and BE values for each case.

**Table 4:** Laboratory findings for patients with spine injuries. INR: International normalized ratio; PT: prothrombin time; PTT: partial thromboplastin time; HCO3: bicarbonate; pCO2: partial pressure of carbon dioxide; BE: base excess. Cells marked with (-) are considered missing data.

Case	INR	PT	PTT	HCO3	pH	pCO2	BE
1	1.3	15.9	25.5	24.7	7.3	50.3	-2.2
2	1.04	13.4	30.8	-	-	-	-
3	1.39	17	32.6	18.6	7.477	27.6	-3.8
4	1.16	14.6	27.7	-	-	-	-
5	1.18	14.7	28.8	-	-	-	-
6	1.41	17.2	27.5	-	-	-	-
7	1.48	17.8	42.5	-	-	-	-
8	0.94	12.4	26.1	-	-	-	-
9	1.02	13.7	29.4	-	-	-	-
10	1.06	14.3	25.3	-	-	-	-
11	1.02	13.8	24.8	-	-	-	-
12	1.31	17.5	27.5	-	-	-	-
13	1.27	17	24.1	20.6	7.37	37.3	3.2
14	1.19	15.9	32	21.6	7.44	32.6	-1.7

Physical examination findings varied, including paraplegia, confusion, facial lacerations, ear bleeding, intubation, limb tenderness, eye swelling, scalp lacerations, back pain, temporomandibular joint (TMJ) dislocation, decreased S3 sensation, and arm abrasions. Imaging requests varied by injury and included CT, MRI, X-rays, and skeletal surveys. Radiology revealed specific injuries: brain edema, spinal fractures (C2, C4-C6, L4), lung atelectasis, spleen laceration, brain contusion, skull fractures, epidural and subdural hematomas, rib fractures, and limb injuries (calcaneus, tibia, mandible). Table 5 summarizes the physical examination findings, ER-type of radiology requested, and radiology findings for each case.

**Table 5:** Radiological findings for patients with spine injuries. CT: computed tomography; MRI: magnetic resonance imaging; TMJ: temporomandibular joint. Cells marked with (-) are considered as missing data.

Case	Physical exam	ER-type of radiology requested	Radio findings
1	Paraplegic	Full CT-scan, chest X-ray, skeletal survey, X-ray spine, MRI SPINE	Mild brain edema. C4-C5 fracture, C5 fracture, C6 fracture
2	Drowsy, confused, multiple face laceration, fresh blood from ears	Full CT-scan	Fractured C2 spine, atelectatic lungs bilaterally, mild spleen laceration
3	Intubated, left leg tenderness	Full CT-scan, Lower limb X-ray	C3-C4 transverse process fracture, left distal ulnar fracture, left-sided hemorrhagic brain contusion with midline shift, parieto-temporal displaced fracture
4	Eye swelling and scalp lacerations	Full CT-scan	Right frontal epidural hematoma with depressed skull fracture and midline shift, T7 compression fracture, T8 burst fracture, T10 inferior endplate vertebral disc fracture
5	Lower back tenderness	Spine CT scan, chest CT scan, abdomen-pelvis CT scan	L1 fracture
6	Low back tenderness	Full CT scan	L4 fracture, bilateral calcaneus fracture
7	Lower back tenderness	Spine CT scan, Lower limb X-ray	Left closed tibial fracture, L3-L4 compression vertebral fracture
8	Free	Head CT scan, spine CT scan, spine MRI	Type 2-3 fracture of the odontoid process of C2, intramedullary hematoma with possible transaction at T4 to T6 levels
9	TMJ dislocation	Full-body CT	Compression fracture of L4 with retropulsion to spinal canal, displaced linear right condylar process of mandible
10	Decreased sensation over S3 distribution	Full body CT	T12 burst fracture, L2 severe burst fracture
11	Left superficial arm abrasion, right upper limb tenderness	Full body CT	Severely slipped ribs (5th, 6th, and 7th), bilateral lung contusion and hemothorax, right occipital minimally displaced fracture, T9-T10 spinous process fracture
12	Free	Full body CT	Left epidural hematoma on parietal region (0.5 cm at max thickness), non-displaced fracture of spinous process C7
13	Free	Full body CT	Diffuse subdural hematoma, partial linear fracture, bilateral subgaleal hematoma, brain edema, linear C7 transverse process fracture
14	Left temporal cut wound	Full body CT	Comminuted fracture of left temporal bone, linear fracture of epicondyle of occipital bone, linear fracture of left transverse process of C2, lung contusion, rib fractures

## Discussion

As head and spinal injuries are associated with high rates of mortality and morbidity, it is important to understand the optimal methods for the management of such injuries to avoid immobilization or any unwanted adverse event [1]. In this descriptive study, which was held in a tertiary hospital, we aim to investigate the different factors that are associated with mortality and morbidity in order to find the optimal therapy whether operative or non-operative, and the best management plan for the patients.

In this study, we included a total of 286 patients who came to the ER suffering from head injuries and 14 patients suffering from spinal injuries. As we conducted this descriptive study for over 10 years, patients had different etiologies and underlying causes. Some of these underlying causes include RTA, falling, or being hit by an object. These are consistent with what is reported by Eggensperger Wymann et al. in addition to violence and sports/playing injuries which are common in older children [11,12].

The different mechanisms of injury were evaluated and assessed across the patients in order to manage the cases accordingly. Some patients were war victims, and the mechanism of injury was a blast, others were blunt or penetrating injuries. Some of these mechanisms suggest opening a wound to further explore, remove foreign bodies, and debride adequately [13].

For children who come to the ER with these injuries, attention should be given to controlling the hemorrhages as the head and spine are reached in blood vessels which may be injured or traumatized as a result [14]. For patients who come with minor injuries, normal imaging, and observation may be undertaken [15,16]. In addition to neurocognitive evaluation which may be altered as a result of the injury. These management methods can be done before discharging the patient and assessed after as an outpatient [17].

For severe cases, management is strict to avoid exacerbations such as brain injury. Maintenance of a normal BP (normotension) is important since patients may have hypoperfusion and hypoxia as a result of systemic hypotension [18]. This can be achieved by using IV fluids for example, and this is how we managed these cases in our emergency department. Initial management as reported by Mtaweh et al. includes intubation for children who suffer from severe traumatic brain injury as a result of head injury and for patients who lost consciousness in which CT scans cannot be used for evaluation [19]. Moreover, avoiding hypothermia, hypoxia, hypotension, and hypercarbia [19]. In our hospital, we managed our patients under these guidelines in which patients either received chest intubation, IV fluids, and/or observation according to the severity of the case.

The blunt and penetrating injuries are associated with a huge risk of vascular trauma. either intracranial or extracranial vascular injuries is a life-threatening condition and should be managed immediately [20]. The most common arteries involved are the cervical carotid artery followed by the vertebral artery. Blunt traumatic injuries to the carotid artery are more common and associated with high mortality rates as reported by Gomez et al. and Biffl et al. [21,22].

A study was carried out in Qatar in which they described and studied falling-related injuries in different age groups and across both genders [23]. They reported that most of these injuries occurred in the middle-aged population followed by pediatrics. The injuries were mostly head injuries and the patients who fell from greater heights had chest and spinal injuries which also required more intensive care and longer hospital stay. For the pediatric age group, falling from heavy objects was more common and thus head injuries were also more common [23].

Spinal injury had different mortality rates which differ according to the spinal region affected. Since the pediatric spine is not fully mature, it is more prone to fractures than the adults’ spine which is more challenging in both evaluation and management [12]. The most affected region is the upper cervical spine in children less than 8 years, and it is more likely to be ligamentous rather than a bone fracture [24]. In older age (> 8 years), the spine begins to fully mature and acquire the adult characteristics in cases of spinal injuries which make the lower cervical spine more commonly affected [25,26].

Leonard et al. reported that mortality and morbidity rates were higher for patients with atlanto-occipital dislocations or dislocations in the first and second cervical spines [25]. Patients with axial injuries had five times increased mortality rates compared to those with sub-axial injuries. An Italian study established a set of guidelines in order to minimize the risk and reduce the mortality rates in those patients [27]. The set included assessments and interventions that should be followed and undertaken in the ER, and these include ensuring breathing and airway, with protection for the cervical spine, controlling hemorrhage and circulation, evaluating the disability and neurologic status, and controlling temperature [27].

Our study was conducted in a tertiary hospital for over 10 years, in which we aimed to assess the patients not only as an in-patient but also follow them over years to see whether certain conditions develop as a result of head or spinal injuries. This study is the first to our knowledge in the Middle East region, that studies how different factors can affect the management modalities and affect the mortality and morbidity rates. A limitation of our study is the low number of patients with spinal injuries which limited our further evaluation and generalization in terms of regions that are associated with higher mortality and morbidity.

## Conclusions

In conclusion, our study reported that early management and diagnosis for head and spinal injuries are the key to reducing the mortality and morbidity rates. The right evaluation and assessment of the cases according to the type, location and mechanism is important to appropriately manage the case and avoid any unwanted events.
